# The rate and spectrum of germline mutations in chicken from a commercial pedigree line

**DOI:** 10.1186/s12711-026-01085-2

**Published:** 2026-09-21

**Authors:** Eugenio López-Cortegano, Bolívar Samuel Sosa-Madrid, Andreas Kranis

**Affiliations:** 1 Aviagen Ltd., Newbridge, Edinburgh EH28 8SZ UK; 2 Aviagen Turkeys Ltd., Tattenhall, Chester CH3 9GA UK; 3 The Roslin Institute and Royal (Dick) School of Veterinary Studies, University of Edinburgh, Easter Bush Campus, Midlothian, EH25 9RG UK

## Abstract

**Background:**

Pedigree-based studies of germline mutations provide a direct understanding of the origin and consequences of genetic variation, and allow quantification of how mutations affect functional variation. Here, we use whole-genome sequencing data from 194 chicken (*Gallus gallus*) trios from a commercial pedigree line to estimate the germline mutation rate and spectrum, and to characterize patterns of mutational variation across the genome and genes, aiming to rank protein coding genes by their expected contributions to mutational variance (*V*_*m*_).

**Results:**

We estimated a mutation rate of *µ* = 3.49 × 10^−9^ mutations per nucleotide site per generation. A goodness-of-fit test (χ^2^_13_ = 10.97, *P* = 0.61) indicated that the number of new mutations per generation follows a Poisson distribution (*λ *= 6.22). A read-based phasing approach revealed a strong sex bias, with males contributing approximately twice as many mutations as females. Microchromosomes showed a point estimate of mutation rate approximately 8% higher than macrochromosomes, likely owing to their higher GC content and a mutation spectrum dominated by C→T transitions. From this spectrum, we built a codon transition matrix and show that genes vary widely in their propensity to mutation based on codon composition, likely reflecting large differences in their contributions to *V*_*m*_. Specifically, we found that genes involved in immunity and development rank among those most prone to non-silent mutations.

**Conclusions:**

Our results reveal predictable patterns of gene-level mutational variation and provide a framework for anticipating how new mutations generate functional variation. By bridging quantitative genetics and molecular biology, we provide a mechanistic basis for variation in *V*_*m*_ across the genome, showing that heterogeneity in *V*_*m*_ can arise from gene-specific features, particularly codon composition and mutational target size. These metrics offer a way to rank genes and prioritize genetic variants by their expected contributions to biological functions as a consequence of *de novo* mutation. This provides a new basis for informed management of genetic variation in breeding populations and for understanding the evolutionary dynamics of functionally important genes.

**Supplementary Information:**

The online version contains supplementary material available at https://doi.org/10.1186/s12711-026-01085-2.

## Background

*De novo* germline mutations are the source of new genetic variation, providing the raw material on which selection acts and thus contributing to evolutionary change. Understanding mutation rates is therefore fundamental across many areas of biology, for example, calibrating molecular clocks [1], interpreting the incidence of human genetic disorders [2, 3], and managing the genetic load in livestock breeding programs [4]. However, accurately estimating these rates is challenging because germline mutations are rare. Early studies relied on mutation accumulation (MA) experiments, in which mutations accumulate in lines of small effective size where selection and recombination are largely ineffective [5]; such designs remain widely used to date (e.g. [6]). Recent advances in DNA sequencing technologies have greatly improved the detection of new mutations [7] and the characterization of their spectra [8, 9], enhancing both the resolution of MA experiments and enabling direct studies in pedigrees and natural populations, where precise mutation rate estimates are critical. These estimates are now available for a wide range of taxa [10, 11], and new studies continue to broaden the range of species analysed (e.g. [12–14]). Beyond their rate of occurrence, a full understanding of the evolutionary impact of *de novo* mutations requires examining the mutation spectrum, i.e., the frequencies at which each type of mutation occurs. The mutation spectrum is not fully random but shaped by processes such as DNA repair efficiency [15, 16] and the epigenetic landscape [17]. Additional biological factors including parental age [18], sex-specific biases [19], and local genomic features such as GC content [20], also influence mutation rates and their spectrum. Environmental mutagens, including exposure to chemicals [21] and ionizing radiation [22], further contribute to variation in germline mutation rates and spectra. Consequently, dissecting the mutation spectrum is essential for understanding the sources of new mutations, predicting their genome distribution, and assessing their contribution to genetic diversity, adaptation, and disease.

Birds represent a compelling clade for studying ecology and evolution [23, 24]. However, direct estimates of their mutation rates remain scarce [25–27], and the limited sample sizes of previous studies constrain our understanding of the distribution of mutations, their spectrum, and potential biases. Avian genomes are characterized by a large number of microchromosomes, which are associated with elevated GC content, gene density, and recombination rates [28], which influence mutation dynamics and the efficacy of selection. For instance, a recent comparative genomic study suggested that variation in evolutionary rates across avian genes is primarily driven by differences in GC content [20]. Other biologically relevant factors, such as age and sex, have also been suggested as key drivers of mutation rate variation among individuals [18, 19]. In birds, a male-biased mutation rate has been inferred from comparisons of Z chromosome and autosome evolution [29]. Similarly, life-history traits such as clutch size and generation length are major predictors of mutation rate variation across the phylogenetic families [20].

Understanding species-specific mutation rates is crucial not only for evolutionary biology but also for applications in conservation genetics and animal breeding. A common genetic challenge in both selective breeding and small populations is the potential for harmful recessive variants to rise in frequency. One notable example is the occurrence of inheritable disorders in dairy cattle, such as bovine leukocyte adhesion deficiency (BLAD) and complex vertebral malformation (CVM), which have been linked to this process [30]. Therefore, accurate mutation rate estimates are crucial, as they can inform strategies to minimise the accumulation of such deleterious alleles, thereby preserving genetic health. As one of the most important livestock species globally [31], the chicken is a prime model for investigating how mutations shape genetic health and production traits. A precise understanding of its mutation rate and spectrum would provide a proactive tool for monitoring and mitigating the genetic load, thereby safeguarding animal health and ensuring the long-term sustainability and productivity of breeding programmes.

Here, we bridge the estimation of the germline mutation rate, a fundamental parameter in evolutionary genetics, with its potential use in animal breeding practice. To do so, we first validated previous estimates of the mutation rate using a large sample (*N* = 342) comprising 194 parent-offspring trios of chicken from a commercial line from the breeding programme at Aviagen (Newbridge, UK). The extensive set of mutations discovered in our study allowed us to characterize the mutation rate by its statistical and genomic distributions, as well as its sex bias and spectrum. Finally, we explored the effects that new mutations have on genomic coding sequences and what biological functions are the most prone to spontaneous changes in function due to mutations. Together, these analyses provide a comprehensive understanding of how new mutations arise in birds, and point to biological processes that are most prone to rapid mutation-driven change.

## Methods

### Biological material, DNA extraction and sequencing

A total of 194 trios with complete information from mother, father, and one offspring coming from a pedigree line of commercial broilers from the Aviagen breeding programme were used in this study, amounting to a total of 342 individuals [Additional file 1, Table S1]. All birds were bred between 30 and 48 weeks of age, with similar ages for sires (mean = 38.3, sd = 5.0) and dams (mean = 37.3, sd = 4.6). DNA was extracted from blood samples using a two-step extraction method from dried blood spots, which included an elution and digestion stage adapted from [32] followed by a final purification with an Invitrogen PureLink Genomic DNA kit. Whole genome sequencing was performed using the Illumina NovaSeq X Plus system at Novogene (Cambridge, UK). The sequencing libraries were generated using a PCR-free approach. This yielded approximately 40× coverage of 150 bp paired-end sequences for each sample.

### Genome assembly and annotation

With the purpose of improving the quality of read mapping to the genome and the discovery of new mutations, we used a combination of long-read sequencing technologies to generate a chromosome-level assembly from a female sample of a commercial chicken line from the Aviagen breeding program. Details on the extraction of high-molecular-weight DNA, long-read sequencing, assembly and annotation are provided in the [Additional file 2, Text S1]. Briefly, long reads from Pacific Biosciences (PacBio) using the circular consensus sequencing (CCS) mode were obtained at 60× coverage and used to create a contig-level assembly draft with hifiasm 0.24.0 [33–35]. With the aid of long reads from Oxford Nanopore Technologies (ONT) at 30× coverage, the contig-level assembly was manually scaffolded to chromosome level based on its mapping to the *Gallus gallus* GRCg7b reference (NCBI accession GCF_016699485.2). The resolved assembly, referred to as Aviagen v1, was highly collinear with GRCg7b [Additional file 3, Fig. S1], while showing higher N50 contiguity (28.2 Mb vs. 18.8 Mb) and gene completeness as assessed by BUSCO v5.8.2 (99.3% vs. 98.6% [36]). Genome annotation was generated by lift-over of the GRCg7b reference annotation (release 106) using Liftoff v1.6.3 [37] with the ‘−polish’ option enabled. To facilitate interpretation of “uncallable” sites (see below, and [Additional file 1, Table S2]), repeat annotation was generated *de novo* using a combination of approaches. Tandem Repeats Finder v.4.09.1 (TRF [38]) was run with parameters “2 7 7 80 10 50 500 -f -d -m -ngs” to identify microsatellite and satellite sequences. In addition, transposable elements (TE) were annotated in two ways. First, with RepeatMasker 4.1.8 (https://www.repeatmasker.org) using the NCBI/RMBLAST v.2.14.1 search engine and the Dfam 3.9 chicken database. Second, running RepeatMasker on a *de novo* TE library generated with RepeatModeler v.2.0.6 [39] and curated with MCHelper v.1.7.0 [40] using the PFAM35.0.hmm library. TE annotations from both approaches were merged.

### Read alignments

Reads were aligned to the Aviagen v1 reference genome using BWA-MEM v0.7.17-r1198 [41]. Individual alignments were then sorted with SAMtools v1.21 [42], followed by read mate-pair information synchronization (“fixMateInfo”), read group replacement (“setReadGroups”), and duplicate marking (“markDuplicates”) using Picard Tools v3.3 [43].

### High-quality genomic regions

We restricted our analysis to new mutations found at high-quality “callable” regions, applying quality thresholds consistent with our mutation-calling criteria (see below). To standardize comparisons across trios, we defined a common callable genome for all samples using stringent filters. Genomic regions were retained only if they met the following criteria:

- Minimum read depth (DP) ≥10 in all samples
- Maximum DP ≤ 80 in all samples
- Read mapping quality (MQ) ≥30 in all samples.
- Non-missing genotype (GT) in ≥99% of samples.

Additionally, we only considered mutations in the nuclear genome, excluding the sex Chromosome W due to its highly repetitive content [44, 45]. DP and MQ values were derived from the alignment files using SAMtools, while GT values were extracted from a joint VCF file generated with GATK 4.6.1.0 [46] (see below). Programs from the BEDTools v2.31.1 [47] and BCFtools v1.22 [48] suites were also used during genomic file processing.

### Identification of de novo mutations, and calculation of the mutation rate

Only single nucleotide mutations (SNMs) were considered. We first used DeepTrio 1.8.0 [49] for mutation discovery, a program built on top of the deep learning-based variant caller DeepVariant [50]. DeepTrio was run with the “WGS” model as recommended for Illumina short reads, and the output GVCF files were merged on a per-trio basis using GLnexus v1.4.1 [51, 52] with the “DeepVariant_unfiltered” configuration preset.

GATK was additionally employed for two purposes. First, to validate SNMs called by DeepTrio, retaining only those identified by both approaches. Second, to generate the genotype information used to define callable regions (see above). To do so, variants were first called from individual samples using GATK HaplotypeCaller with “-emit-ref-confidence BP_RESOLUTION” to include both variant and invariant sites. We then used CombineGVCFs and GenotypeGVCFs (with the option ‘-include-non-variant-sites’) to merge all samples and genotype calls at nucleotide resolution. Genotype posterior probabilities were calculated using pedigree information with the GATK tool CalculateGenotypePosteriors (options ‘–pedigree’ and ‘–skip-population-priors’).

To determine the parent-of-origin of new mutations, we phased them using the read-phasing algorithm implemented in WhatsHap 2.8 [53–55]. To do so, we used BCFtools to extract each new mutation from the GATK-generated VCF file and all variants 300 bp upstream and downstream from it together with the genotypes for all samples from the trio where the mutation was discovered. Since phasing cannot be determined by new mutation alone, we first used the child genotypes to determine the haplotype in which the mutation occurred, and then assigned probabilities to each parent based on their count of haplotypes matching the child’s. We validated the parent-of-origin of phased mutations manually by visualising read alignment files using the Integrative Genomics Viewer v2.19.1 [56] (IGV).

Each of the 194 trios analysed was uniquely identified by a single child and two parents. New mutations were counted only in the child, as variants present in the child but absent in both parents. Mutations required a minimum quality score (QUAL) of 30 on the Phred scale, a read depth between 10 and 80, and non-missing genotype information for all of the samples in the trio. Additionally, variant sites were required to be biallelic. A total of 1409 mutations passing these filters were manually validated by visualising the trio alignment files with IGV, of which 1206 (~85%) were confirmed. Rejected candidates generally showed lower read depth (mean DP ≈30×) and visual evidence of alternate alleles in one or both parents, often occurring in phase on the same haplotype, consistent with parental mis-genotyping in potentially paralogous regions that passed the DP < 80 filter.

The per haploid genome per generation mutation rate (*U*) was estimated as *U*_*i*_ = *N*_*i*_ / 2, where *N*_*i*_ denotes the count of mutations found in individual *i*. The per nucleotide site per generation mutation rate (*µ*) was derived as *µ*_*i*_ = *U*_*i*_ / *N*_*c*_, where *N*_*c*_ is the number of callable sites. When calculating *µ*_*i*_ at specific regions, the values of *N*_*i*_ and *N*_*c*_ were adjusted accordingly.

### Statistical analysis

Statistical analyses were performed in R v4.4.1 [57]. To calculate the fit of the mutation rate to a Poisson distribution, we fitted the count of mutations per individual chicken to a Poisson distribution using the R package fitdistrplus v1.2.4 [58]. Following the absence of a detectable association between the number of mutations and parental age (see Results), we performed a post hoc simulation-based power analysis using the 194 trios in the study. For this analysis, we generated 1000 replicate datasets by resampling parental ages with replacement from the observed distribution. Synthetic mutation counts were then generated as a linear function of standardized parental age with added random noise, where the magnitude of the association was controlled by an assumed correlation coefficient *r*, and the residual variance was estimated from the observed data. For each assumed value of *r*, statistical power was calculated as the proportion of replicate datasets yielding a significant association (*p* < 0.05). The minimum detectable correlation was defined as the smallest value of *r* for which statistical power reached 80%. An R script supporting the main mutation analyses is included as [Additional file 4, Code S1].

### Predicted mutation effects

The SNM spectrum shows the distribution of different types of SNMs. To assess the influence of the SNM spectrum on mutations in protein-coding sequences (CDS), we used the dedicated script “expLoadSNM” [59]. Briefly, the pipeline follows three steps: (1) 10,000 synthetic point mutation sites are randomly assigned in CDS of the callable chicken genome. (2) For each mutation site, the ancestral nucleotide determines the three possible substitution types (e.g., C→A, C→G or C→T for a C site), and the actual substitution is then randomly realized according to their relative probabilities in the SNM spectrum (e.g., C→A transversions have a probability *P*_C→A_ that is given by the SNM spectrum). Alongside the nucleotide substitution type, amino acid substitutions are also annotated in this step. (3) The resulting mutations have their effects predicted using SnpEff v5.1 [60], which annotates synonymous, missense and nonsense mutations with low, moderate and high effect sizes, respectively. The pipeline was run for 1000 replicates.

As a result of these simulations, we obtain a matrix with the probabilities of each substitution type to produce a given effect (e.g., low, moderate or high) when occurring at a given amino acid. We denote this probability as *P*_S,E | AA_ for a given substitution *S*, effect size *E* and amino acid *AA*. For example, *P*_A→C,low | Ala_ denotes the probability that an A→C substitution occurring at an alanine codon produces a mutation of low effect. This matrix thus provides a means of scoring CDS according to their susceptibility to experience functional changes from new mutations. It should be noted that equivalent empirical probabilities could also be derived directly from the SNM and codon spectra information. However, since the chicken genome annotation was used, our matrix included not only amino acid change probabilities but also start codon loss, which otherwise requires codon positional information.

Using these probabilities, we then calculated mutation effect probabilities for each amino acid, i.e., *P*_E | AA_, from the sum of marginal probabilities *P*_S,E | AA_ for each substitution type. For example, alanine-encoding codons have a low-effect mutation probability *P*_*low* | Ala_ = 0.21 that results from summing the marginal probabilities *P*_A→C,low | Ala_ + *P*_A→G,low | Ala_ + *P*_A→T,low | Ala_ + *P*_C→A,low | Ala_ + *P*_C→G,low | Ala_ + *P*_C→T,low | Ala_. Using these probabilities, we define a predicted rate-integrated mutational effect (PRIME) score to rank proteins in the chicken genome according to their risk of moderate (*PRIME*_*M*_) or high (*PRIME*_*H*_) effect mutations. For protein with *L* amino acid-encoding codons, the score was calculated as:

$$PRIM{E_E} = \mathop \sum \limits_{AA = 1}^L {P_{E |AA}}$$

In all cases, this score increases with CDS length, as longer sequences present a larger mutational target. To correct for protein length, *PRIME* can be divided by *L* yielding a length-normalized score that depends only on the specific codon composition of the protein sequence.

This score can be interpreted in terms of the underlying mutational variance (*V*_*m*_ [61]) since each probability ${P_{E |AA}}$ can also be expressed as a sum over the codon’s three nucleotide (*N*) positions and all three possible substitutions *S*:

$$\mathop \sum \limits_{AA = 1}^L {P_{E |AA}} = \mathop \sum \limits_{AA = 1}^L \mathop \sum \limits_{N = 1}^3 \mathop \sum \limits_{S = 1}^3 {\mu _{AA,N,S}} \,\alpha _{AA,N,S}^2$$

Where *α* here denotes the effect size of mutations, but not in the classical sense from quantitative-genetics sense of an allelic substitution effect; rather, it is a variable quantifying the functional effect assigned to each mutation. Here, we assume *α = 1* for non-silent mutations and *α = 0* otherwise. Despite these differences in the scale of *α*, we expect *PRIME* and *V*_*m*_ to be correlated to the extent that non-silent mutations cause phenotypic changes. Therefore, *PRIME* can be used as a practical metric for ranking genes by their expected contributions to *V*_*m*_. However, *PRIME* is expected to take numerically much larger values than *V*_*m*_ because allelic substitution effects are usually very small; in practice, their exact value is also often unknown.

### Gene ontology (GO) and enrichment analysis

To categorize the biological functions of genes of interest, we retrieved their GO terms using biomaRt v2.62.1 [62, 63]. Since many chicken proteins lacked GO annotations in the Ensembl database (“ggallus_gene_ensembl”, release 115, April 2026) we first identified their human orthologs and then extracted the corresponding GO terms from the human database (“hsapiens_gene_ensembl”). We performed GO enrichment analysis on the resulting terms using the “enrichGO” function from clusterProfiler v4.14.6 [64], focusing on the “biological process” ontology (‘ont = “BP”’).

## Results

### Mutation rate variation

**Fig. 1:**
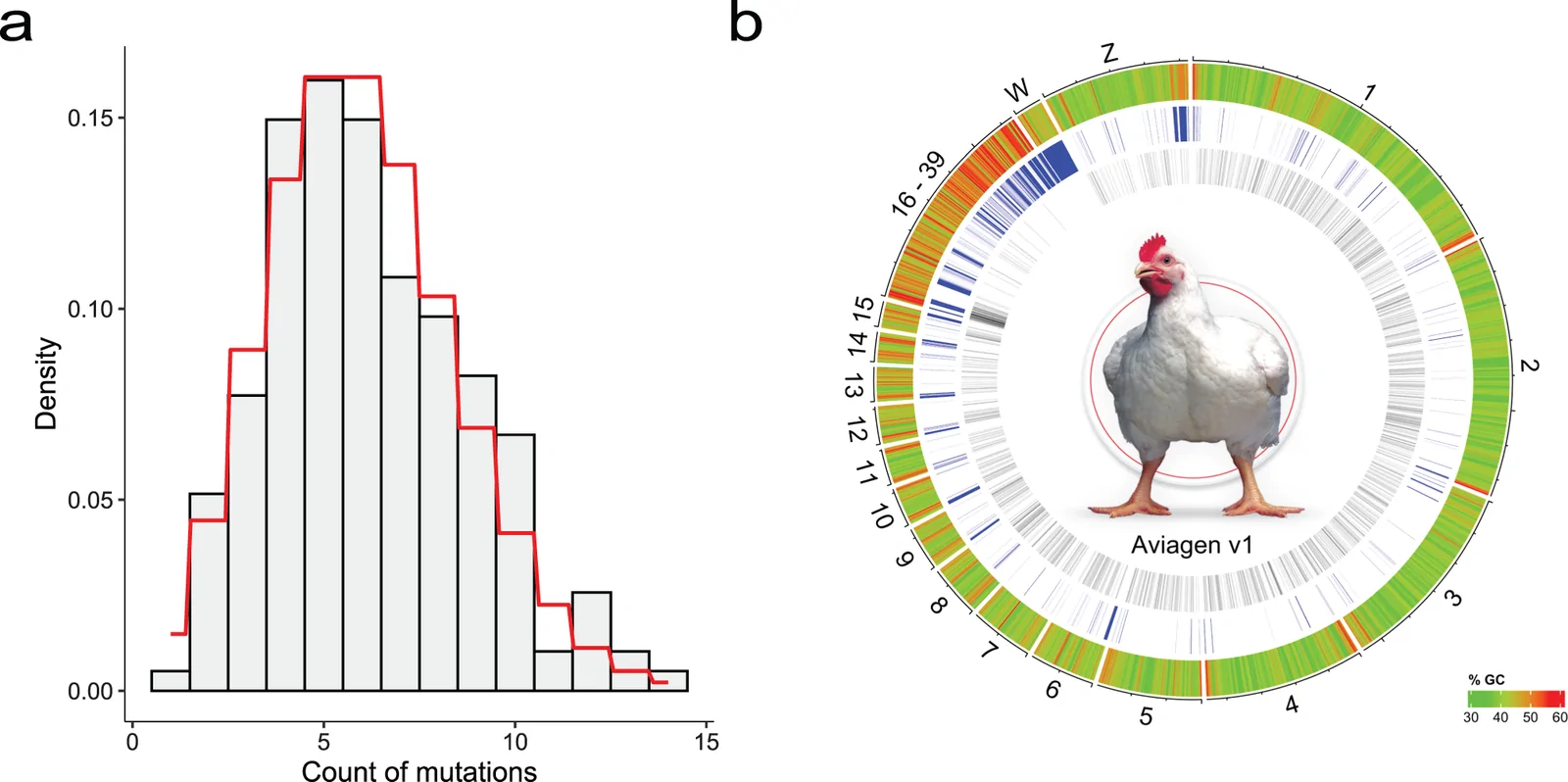
Distribution of mutations. (**a**) Histogram showing the density for the count of mutations among 194 individuals. A red line shows the expected density of a Poisson distribution with rate parameter λ = 6.2. (**b**) Circos plot (Krzywinski et al. 2009, Gu et al. 2014) showing the genome-wide distribution of SNMs. Each sector represents a chromosome (20 Mb per tick); micro-chromosomes 16–39 are shown as a single concatenated sector. Moving inwards from the outermost track, the plot shows: (1) %GC content (green-to-red colour scale); (2) uncallable genomic regions longer than 30 kb (in blue rectangles), with unannotated regions corresponding to callable portions of the genome; (3) individual SNMs (in black rectangles)

We analysed whole-genome sequencing data from 194 chicken trios (342 individuals total) from a commercial pedigree line. From the 1.1 Gb nuclear genome, we identified 869.8 Mb (~80%) as high-quality sites, amounting for a total of 297.5 Gb of sequencing data across all trios. We found 1206 single nucleotide mutations, resulting in a median mutation rate of *U* = 3 SNMs per haploid genome per generation (*µ* = 3.45 × 10^−9^ per nucleotide site per generation). The count of mutations per individual suggests only minimal overdispersion compared to the expectation based on a Poisson distribution (mean = 6.2, variance = 6.7, Fig. 1a), and a goodness-of-fit test did not reject a Poisson model with rate parameter λ = 6.2 (χ^2^_13_ = 10.97, *p* = 0.61). All detected mutations are listed in [Additional file 1, Table S3].

All mutations found were heterozygous, except for hemizygous mutations in the Chromosome Z of females. Among the phased autosomal mutations that could be unambiguously assigned to parental inheritance (*N* = 323), two thirds were derived from the male parent (66.6%) implying that males contribute twice as many mutations to their offspring compared to females. This inheritance pattern persisted when analysing all mutations with phasing information (*N* = 398) using probability-weighted assignments based on paternal haplotype support. In contrast, a similar number of mutations was observed to be inherited in the male and female offspring (Wilcoxon test, *W* = 5268.5, *p* = 0.07). This near-significant trend may reflect the inclusion of the Chromosome Z, as males inherit two copies whereas females inherit one. Consistent with this interpretation, restricting the analysis to autosomes removed this pattern (*W* = 5019.5, *p* = 0.25). No correlation was observed between parental or maternal age and the number of mutations inherited (Pearson’s product-moment correlation, *t*_192_ < 0.4, *r* < 0.03, *p* > 0.68). A post hoc power analysis indicated that, given the sample size and breeder age distribution in this study, we had 80% statistical power to detect correlations of approximately *r* > 0.5. Thus, while no association was detected, our data suggest that any effect of parental age on mutation number within the sampled age range (30–48 weeks) is likely to be smaller than this threshold. However, smaller effects, or effects that become apparent across a broader range of parental ages cannot be excluded.

### Genomic distribution of SNMs

SNMs were distributed genome-wide (Fig. 1b). We found mutations in all macrochromosomes (Chromosomes 1–9 and Z) and in 22 of the 30 microchromosomes. The absence of mutations in some microchromosomes can be explained by their smaller mutational target and reduced proportion of callable sites. Microchromosomes collectively represent only ~22% of the genome, with some of them contributing as little as 0.0198% (Chromosome 39). While macrochromosomes had an average of 87.9% callable sites, this proportion decreased to 59.9% for microchromosomes, some of them containing as little as 4857 bp (0.136%) of callable sites (Chromosome 37). The lower callable rate of microchromosomes is probably a reflection of their higher GC content (Fig. 1b) and the use of short-read sequencing, which is biased against GC-rich sequences [65].

Measured on a per-chromosome basis, including microchromosomes with no detected mutations, the median mutation rate was *µ* = 3.58 × 10^−9^. Mutation rates across individual chromosomes showed substantial variation, particularly among microchromosomes (from *µ* = 0, to *µ* = 3.73 × 10^−8^), most likely reflecting sampling error due to their small size. When all 144.6 Mb of callable sites from microchromosomes were grouped, their mutation rate was *µ* = 3.81 × 10^−9^. This estimate was slightly higher (~8%) than observed for macrochromosomes (*µ* = 3.53 × 10^−9^). However, this difference should be interpreted cautiously, as the 95% bootstrap confidence intervals (CIs) overlap between microchromosomes of ([3.32, 4.46] × 10^−9^) and macrochromosomes ([3.19, 3.82] × 10^−9^) based on 1000 replicates generated by resampling chromosomes with replacement.

**Fig. 2:**
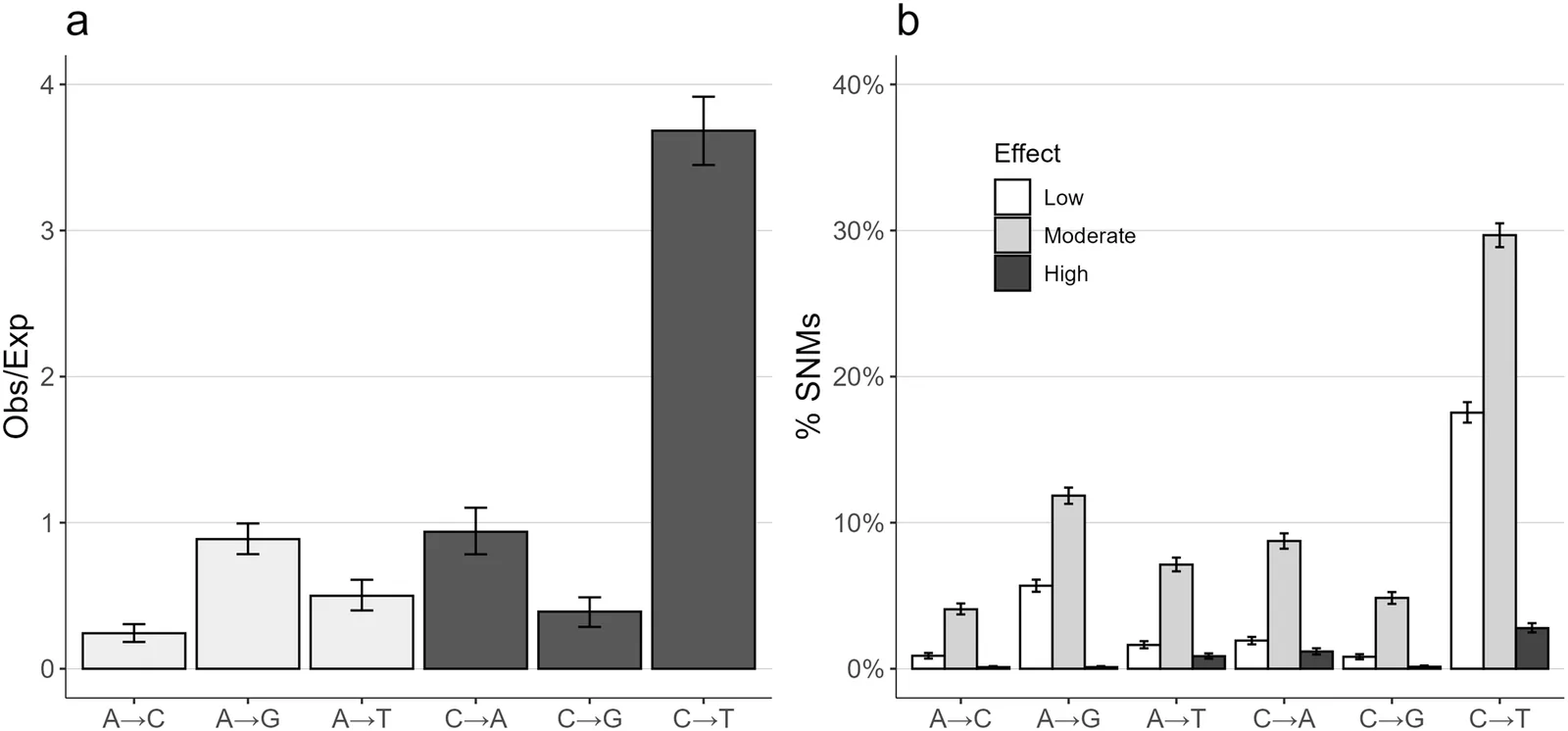
SNM spectrum. (**a**) GC-adjusted ratio of observed vs. expected SNMs. The height of the bars represents the deviation of the observed value from the expectation for all SNMs being equally frequent, after correcting for GC content at callable sites (40.5%). Light bars refer to mutations at A:T sites, whereas dark bars refer to C:G sites. Error bars show 95% CIs based on 1,000 bootstrap replicates. (**b**) Proportion of SNMs predicted to have low, moderate or high effect sizes, based on computer simulations. Error bars show 95% CIs based on 1000 simulation replicates

The distribution of inter-mutation distance (IMD) revealed a small number of clustered mutation events, with seven SNM pairs occurring within 100 bp [Additional file 3, Fig. S2]. In contrast to the overall SNM spectrum (Fig. 2a), most mutations within these clusters occurred at A:T sites, and four involved A→C transversions, the rarest SNM class observed in the full dataset. This shift in mutational spectrum suggests that clustered mutations may arise through mechanisms distinct from those responsible for the majority of SNMs. Possible biological explanations for this deviation in the SNM spectrum include DNA polymerase errors [66] or unrepaired oxidative damage, as 8-oxoguanine is associated with C↔A transversions [67]. We also note that distinguishing true clustered *de novo* mutations from residual bioinformatic artefacts remains challenging, and that a small number of clustered candidates may reflect sequencing, mapping, or variant calling errors despite our stringent filtering and manual inspection efforts.

In terms of their functional annotation, most mutations occurred in intronic (54.5%) and intergenic regions (34.5%), with only a small fraction of SNMs located at coding sequences (CDS, 4.1%) and gene untranslated regions (UTRs, 6.9%). These proportions were in broad agreement with the expectation based on the simulation of synthetic point mutations at genomic callable sites [Additional file 3, Fig. S3], and suggest similar mutation rates across genic and intergenic regions, in agreement with a previous study in the passerine *Acrocephalus arundinaceus* [27]. Among the 49 SNMs found in CDS, the majority (63.3%) of them were missense mutations, followed by synonymous (30.6%) and nonsense mutations (6.1%).

### SNM spectrum and predicted effects

On average, each chicken inherits 6.2 SNMs per generation, half of which (*N* = 3.1) are C→T transitions. The remaining substitutions occur at lower frequencies: A→G (*N* = 1.1, 17.7%), C→A (*N* = 0.7, 11.9%), A→T (*N* = 0.6, 9.6%), C→G (*N* = 0.4, 5.8%), and A→C (*N* = 0.3, 5.1%). However, it should be noted that the chicken genome has a GC content of 42.5%, meaning AT present a larger mutational target. In callable sites, the observed GC content was slightly lower (40.5%). To account for this, we calculated the ratio of observed versus expected SNM frequency, where the expectation is derived from the GC content. Under this model, each of the three possible SNM types at A:T sites has a probability to occur of 0.595 × 1/3 ≈ 0.198, while mutations at C:G sites are expected at a frequency of 0.405 × 1/3 = 0.135. The adjusted SNM spectrum (Fig. 2a) exhibits a strong C→T bias (3.68), common to many other organisms [10], and a higher-than-average mutation rate only for this type of nucleotide substitution (*µ*_*C→T*_ = 4.41 × 10^−9^, [Additional file 5, Table S4]. No substantial differences were observed between the spectra of male- and female-inherited SNMs [Additional file 3, Fig. S4]. A regression analysis revealed no association between the count of inherited mutations and excess of specific SNM types (*p* > 0.08), suggesting similar SNM spectra across individuals regardless of mutation burden.

To explore the expected effect sizes of mutations in the chicken genome, we conducted a simulation study by introducing synthetic SNMs into coding sequences and annotating their expected effects using SnpEff [60]. This software annotates mutations with “low”, “moderate” or “high” effect sizes for synonymous, missense and nonsense mutations, respectively. Simulations were necessary for this exercise due to the limited number of mutations found in CDS in our study (*N* = 49). [Additional file 6, Table S5] includes a transition matrix with probabilities of changes among all amino acid types. Owing to their high frequency, C→T transitions contributed the most in absolute terms to moderate (44.7%) and high (53.7%) effect mutations per generation (Fig. 2b). We expect that the majority of high-effect C→T mutations occur in glutamine (59.4%) and tryptophan (30.8%) codons. Other important sources of high-effect mutations were C→A (22.8%) and A→T (16.6%) transversions, predominantly affecting codons for glutamic acid (50.4%) and lysine (44.7%), respectively. These findings allow us to infer what genes are more prone to functional changes due to mutation. Some examples are provided below.

### Gene-level variation in mutational target and effect

We used data from the SNM spectrum and the simulations described above to score 13,256 protein-coding genes in the chicken genome based on their likelihood of accumulating codon-altering mutations, with either of moderate (*PRIME*_*M*_) or high (*PRIME*_*H*_) effect [Additional file 7, Table S6]. As expected, both scores strongly correlated with CDS length (*t*_13254_ > 664, *r* > 0.98, *p* < 2.2 × 10^−16^), reflecting the larger mutational target of longer coding sequences. The *titin* (*TTN*) gene, encoding a filamentous protein essential for muscle and cardiac function, emerged as a clear outlier due to its exceptionally long CDS (~33 kb). After correcting for CDS length, only codon composition influenced the expected mutational effects. Length-corrected scores showed that longer genes tended to have intermediate probabilities of amino acid changes, while the impact of new mutations was most variable for the shortest peptides [Additional file 3, Fig. S5].

Using length-normalized *PRIME*_*M*_ scores also highlighted structural proteins as key targets of mutation-driven change. For example, 16 out of 143 (11.2%) upward outliers belonged to the collagen (*COL*) family. A GO enrichment analysis, using human orthologs to leverage their more robust functional annotation, revealed strong associations with tissue development and organization as well as detoxification processes [Additional file 3, Fig. S6]. In contrast, length-normalized *PRIME*_*H*_ outlier genes were enriched for immune response regulation, cellular respiration, and sensory perception [Additional file 3, Fig. S6]. No genes overlapped as upward outliers for both length-corrected *PRIME*_*M*_ and *PRIME*_*H*_ scores, but nine genes were downward outliers for both metrics. These included essential energy metabolism genes: five from the NADH dehydrogenase (*ND*) family, two from the ATP synthase (*ATP*) family, cytochrome B (*CYTB*), and the *LASP1* Neighbor (*LASP1NB*) gene.

## Discussion

Using data from 194 chicken trios, we present the largest study to date estimating the *de novo* germline mutation rate in any bird or livestock species. Our estimated per-nucleotide site per-generation SNM rate *µ* = 3.45 × 10^−9^ is in reasonable agreement with previous estimates in chicken (*µ* = 3.63 × 10^−9^ [26]), and falls within the range observed for other bird species [25–27]. Given that chickens breed approximately once per year, this rate also aligns broadly with phylogenetic estimates based on between-species divergence (1.2–4.0 × 10^−9^ substitutions per year [68–70]). The agreement of our estimate with previous chicken and avian mutation rate estimates supports the idea that sustained artificial selection in the commercial pedigree lines analysed here has not resulted in physiological or genomic changes leading to a substantially elevated mutation rate, such as the fixation of mutator alleles as observed in some inbred mutation accumulation lines (e.g., the FVB mouse strain [59, 71]). However, because all individuals in this study originated from a single commercial pedigree line with a history of intensive selection, caution is warranted when extrapolating these estimates to *Gallus gallus* more broadly. Additionally, our study was limited to breeders aged 30–48 weeks, whereas chickens can reproduce over several years. As parental age has been associated with increased germline mutation rates in both mammals [26, 72] and birds [26], the extent to which our estimate applies across the full reproductive lifespan of chickens remains unclear. More generally, despite our extensive filtering efforts, a small number of false-positive and false-negative mutation calls may remain, as it may be expected from the inherent challenges in detecting germline mutations [7]. Nevertheless, because our mutation rate estimates are based on the median across a large number of independent trios, any residual errors are unlikely to meaningfully affect the overall mutation rate estimate.

Compared to other livestock species, chickens exhibit mutation rates at the lower end of the spectrum (*µ* = 3.6–12.0 × 10^−9^ [4, 26, 73, 74]). Two factors may explain this. First, a larger ancestral effective population size (*N*_*e*_) in chickens could have reduced mutation rates due to more efficient selection against deleterious mutator alleles, following the drift-barrier hypothesis [75, 76]. Supporting this idea [77], estimated an ancestral *N*_*e*_ for the genus *Gallus* of at least 1 million, whereas estimates for other, mammalian livestock species are substantially lower [74, 78]. Second, chickens’ younger reproductive age may contribute to their comparatively low mutation rate.

A male-to-female mutation bias has long been hypothesized in birds [29, 69, 79], and recent pedigree-based studies of the mutation rate provide further support for this phenomenon [26, 27]. By inferring the parent-of-origin for 323 SNMs, our results offer strong evidence for a substantial sex bias in the mutation rate of chickens, with males contributing nearly twice as many mutations as females to their offspring. This finding agrees with a previous estimate of male-to-female mutation bias α = 1.8 in birds [29]. A similar, though typically larger, bias is well-documented in mammals [29]. Both sex and age effects are well-established drivers of mutation rate variation, with rates generally higher in males and increasing with parental age (e.g., humans [18]). Our study, which was powered to detect correlations larger than *r* > 0.5 between parental age and the number of inherited mutations, did not find significant associations. While this could suggest a weaker parental age effect on mutation rates in birds than in mammals [18], this likely reflects the young and narrow breeder age range in our sample (30–48 weeks), which limits the ability to resolve age-related effects on mutation rates. A broader range of breeder ages will be required to quantify the contribution of parental age to mutation rate variation in chickens. In mammals, differences in DNA-repair mechanisms have been proposed as a key factor underlying male-biased mutation rates and the higher rate of paternally-inherited mutations [19, 80]. However, our results advise caution in directly extending this explanation to birds. Alterations in DNA-repair pathways are often linked to changes in the mutational spectrum [66, 81], yet we observed similar mutational spectra of male- and female-inherited mutations [Additional file 3, Fig. S4]. This suggests that a different mechanistic explanation may be responsible for the observed sex bias in birds, such as differences in germline replication.

Avian microchromosomes are GC-rich (Fig. 1b[82]), and a higher mutation rate compared to macrochromosomes has been inferred in previous studies based on nucleotide substitution rates (e.g. [20, 83]). From the higher mutation rate at GC sites observed in the chicken SNM spectrum, we expect approximately an 11% higher mutation rate in microchromosomes compared to macrochromosomes, based on their different GC content at callable sites (50.3% vs. 40.2%), with substantial variation in the expected mutation rates among chromosomes [Additional file 3, Fig. S7a]. This result is in broad agreement with our observation of mutation rates in microchromosomes being higher than in macrochromosomes, with a point estimate of ~8%, and also with a recent study identifying GC content as the main driver of mutation rate variation across avian genomes [20]. With a higher mutation rate in microchromosomes, particularly C→T transitions, their high GC content is likely the result of high recombination rates and biased GC gene conversion in these chromosomes [28], as well as intense natural selection maintaining high GC content, since their expected GC content at mutation equilibrium is only 20%. Based on our simulation results, there were only marginal differences between the expected effects of mutations in macro- and microchromosomes [Additional file 3, Fig. S7b,c], suggesting that differences in mutation rate underlie most differences in their mutational load. However, it is interesting to note an overall reduction in the relative proportions of both nonsynonymous (from 66.4% to 64.3%) and nonsense (from 5.1% to 4.6%) mutations in microchromosomes [Additional file 3, Fig. S7b,c]. Microchromosomes are gene-dense and host genes with housekeeping functions [84, 85], and we hypothesise that they have evolved codon compositions more robust against deleterious substitutions.

To estimate the expected contributions of new mutations to genetic variance, we derived the *PRIME* score, which is contributed to by three factors. First, the length of the coding sequence, represented by a summation term. The second factor is the nucleotide composition of the DNA sequence, represented by the mutation rate term, resulting in higher values for more GC-rich coding sequences. As a first-order approximation, this term captures broad compositional effects on mutation probability, although mutation rates vary with sequence context and may be higher at specific regions (e.g., CpG sites, [Additional file 3, Fig. S8]). Finally, the third factor is the codon composition itself, represented by the probability of the encoded amino acid to change following a mutation event. Understanding the effect of mutations on genes as a function of their molecular composition is important because it allows inference of mutation-driven evolutionary rates. The mutational variance (*V*_*m*_) is a key parameter in quantitative genetics, measuring the amount of new phenotypic variation arising each generation due to mutation [86]. It is responsible for regenerating standing genetic variance, the fuel of selection [87, 88]. Similarly to additive variance, *V*_*m*_ critically depends on the effect size of mutations and their distribution [89]. Consequently, genes with higher *PRIME*_*H*_ and *PRIME*_*M*_ scores can be inferred to contribute more to *V*_*m*_, either owing to their large mutational target or their codon composition. The high correlation observed between *PRIME* and CDS length suggests that CDS length is a major driver of variation in the value of *PRIME*, and presumably of mutational variance (*V*_*m*_) owing to the larger mutational target. This positions structural proteins – such as those involved in the cytoskeleton, axonogenesis, and muscle development – as a group particularly prone to change under new mutations, with the clearest example being the large *TTN* gene, involved in muscle and cardiac function, including several myopathies [90, 91].

To isolate the effects derived from the nucleotide and codon sequence, the length-corrected *PRIME* score can be used. It should be noted that this regularized metric no longer reflects an expectation to rank genes by their contribution to *V*_*m*_, but nevertheless informs on the probability of mutations having a certain effect size on CDS. We inferred that specific genes – and biological functions – are more prone to change as a consequence of mutation, owing to their distinct codon compositions. Codons encoding tryptophan (Trp) and glutamine (Gln) are expected to have the highest effect sizes under an SNM spectrum biased towards C→T transitions. Accordingly, we found that proteins from genes identified as upper outliers for the length-corrected *PRIME*_*H*_ score were enriched for these amino acids compared to the genomic background (Trp: 2.41% vs. 1.19%; Gln: 8.07% vs. 4.27%). Examples of such proteins include genes from the major histocompatibility complex (MHC) involved in immunity, such as the c-type lectin-like 17 (*YLEC17*; Trp: 7.55%) gene, and others like the forkhead box P2 (*FOXP2*; 16.5% Gln) transcription factor, well known for its role in vocal learning and behaviour [92]. Similarly, proteins with high *PRIME*_*M*_ scores showed a distinct codon composition (e.g., ~2× enrichment of proline and glycine, and depletion of tryptophan, leucine, and isoleucine). More specifically, GC-rich proteins are densely packed with C→T transition sites and may experience more mutations, resulting in higher *PRIME*_*M*_values. One remarkable example is the collagen (*COL*) family, characterized by repeats of glycine and proline triplets [93]. Our results therefore suggest substantial variation in *V*_*m*_ across genes, in agreement with previous studies measuring *V*_*m*_ for gene expression in model species [94–101]. Our results also point to a faster regeneration of genetic variance for genes involved in biological functions such as development and immunity, possibly offering sustained opportunities for adaptive evolution and selection. It should be noted, however, that our study relied on the use of human orthologs for the functional enrichment analysis, and therefore differences between annotation versions and orthology assignments may vary with database updates, highlighting the need for improved annotation in chicken.

From a commercial breeding perspective, our results suggest that chickens generate a substantial number of new mutations each generation. Following our results, approximately 171 codon-altering SNMs are expected to arise per generation in a small farm of 1000 birds. Given that traits of economic importance, such as muscle growth and immune function, are among those predicted to be most affected by mutation, these figures point to a substantial potential to capture and utilize novel genetic variation as it arises. At the same time, failure to monitor and manage new deleterious mutations carries risks, as such variants may drift to high frequencies (and fixation) in populations with low effective sizes.

Since *V*_*m*_ accumulates over time, it can be captured by (G)BLUP approaches when used in combination with individual phenotypes [89]. Knowledge of genes contributing differentially to *V*_*m*_ may therefore help prioritize markers for future genotyping panel designs, although the practical utility of this approach remains to be demonstrated empirically. The low linkage disequilibrium between rare mutations in early generations after their emergence means that standard genotyping arrays have limited use in the short term to prevent the spread of deleterious mutations. More generally, it remains challenging to validate *PRIME* using existing resources such as QTL datasets, as these reflect segregating variation shaped by selection and drift, whereas *PRIME* captures the expected contribution of newly arising functional variation, much of which may be transient due to drift or removed by selection if deleterious. Consistent with this, we found no correlation between gene *PRIME* scores and their QTL count using data from the AnimalQTLdb [102] (release 59) (Pearson’s product-moment correlation, *t*_2420_ > −0.38, *r* ≈ −7 × 10^−3^, *p* > 0.70). We interpret this result cautiously, as genes experiencing more mutations could simply experience higher rates of variant loss by drift or selection. More direct validation may therefore require experimental systems such as mutation accumulation lines, particularly when combined with recombinant lines to assess the effect of individual mutations [103]. Because PRIME is currently defined using coding sequences, species with compact genomes may provide especially suitable systems for such validation. Nevertheless, extending the framework to incorporate effects of mutations in regulatory genes on their expression remains an important avenue for future research. For the near future, periodic sequencing approaches (e.g., genotype-by-sequencing) may provide a more direct means to detect and monitor new mutations that escape array-based detection. This strategy can be combined with ongoing breeding practices aimed to target a rate of inbreeding to below 1% (*N*_*e*_ = 50) as an accepted guideline [104]. Ultimately, cost-effective strategies to monitor mutational input will be essential to balance continued genetic progress with the long-term maintenance of population health.

## Conclusions

Our results demonstrate that the germline mutation rate in chickens is shaped by genomic context (e.g., GC content) and biological factors (e.g., sex). The *PRIME* score quantifies the potential of different genes to generate functional variation through new mutations (i.e., *V*_*m*_). This knowledge provides a framework for anticipating the introduction of functional variation in breeding populations, informing strategies to balance genetic progress with the long-term maintenance of genetic variation. Ultimately, integrating mutation rate estimates and spectrum analysis into genomic selection programs can enhance decision-making in animal breeding and support the sustainable management of genetic resources. Beyond applied breeding, these findings provide insight into the evolutionary dynamics of gene-level variation and the factors shaping mutational input in natural populations.

## Electronic supplementary material

Below is the link to the electronic supplementary material.

Supplementary Material 1

Supplementary Material 2

Supplementary Material 3

Supplementary Material 4

Supplementary Material 5

Supplementary Material 6

Supplementary Material 7

## Data Availability

The whole-genome sequencing dataset generated is proprietary to the Aviagen Group, and is not publicly available due to commercial confidentiality. Access to the data may be granted to qualified researchers upon reasonable request to the corresponding author, subject to a formal data sharing agreement and permission from the Aviagen Group.
