# Supplementary material for: The rate and spectrum of germline mutations in chicken from a commercial pedigree line
Source: Genet Sel Evol. 2026 Sep 21;58:70. doi: 10.1186/s12711-026-01085-2 (PMC13591848; doi:10.1186/s12711-026-01085-2)
Supplement: Supplementary file 1 [file 12711_2026_1085_MOESM2_ESM.pdf]

Text S1. Chromosome-level genome assembly

DNA sequencing

Long-reads were used to build a genome assembly. High-molecular-weight (HMW) DNA was extracted from 1.5 g of breast muscle tissue from a single female and sequenced on a pacific Biosciences (PacBio) Sequel II platform at Hudson Alpha (Huntsville, Alabama, U.S.A.). This generated 60 × coverage of HiFi reads using the circular consensus sequencing (CCS) mode. To supplement the assembly, we also sequenced the same individual on an Oxford Nanopore Technologies (ONT) platform for 30 × coverage, and an Illumina platform for 60 × of Illumina coverage of paired-end reads.

Genome Assembly

A first genome assembly draft was generated *de novo* at the contig-level with the software hifiasm 0.24.0 (Cheng et al. 2021, 2022, 2024) using PacBio HiFi reads. Additionally, a second contig-level assembly was generated from ONT reads by enabling the dedicated option ("--ont") in hifiasm. Given the higher contiguity of the assembly obtained with PacBio HiFi reads (see below), this assembly was used as backbone for scaffolding while the ONT assembly was used to close assembly gaps (see below). The ONT assembly was further polished using Illumina reads, following the ONT polishing pipeline (https://github.com/nanoporetech/ont-assembly-polish). Essentially, the software pilon 1.24 (Walker et al. 2014) was run to fix single nucleotide and indel variants ("--fix bases"). Unless otherwise stated, below we refer to the primary assembly generated with PacBio HiFi reads.

Manual scaffolding

To build a chromosome-level reference genome, the raw assembly was filtered to retain only contigs longer than 30 kb with an average read depth of more than 5 reads. This step removed 67 out of 464 contigs (14.4%). Next, the assembly was scaffolded to the chromosome level by mapping against the *Gallus gallus* GRCg7b reference (with NCBI accession GCF_016699485.2). Mapping files were generated with minimap2 2.24 (Li 2018, Li 2021), considering only entries with mapping lengths longer than 30 kb and high mapping quality (MQ ≥ 60). The mapping of contigs was validated with Mashmap 3.1.3 (Jain et al. 2018). Only contigs mapping assembled chromosomes in the GRCg7 reference were considered. Assembly gaps were filled with 10 kb sequences of unknown 'N' nucleotides. Additionally, a mapping of the ONT assembly allowed to close a total of eight gaps (10%) with sequences derived from the polished ONT assembly. During the scaffolding process, the assembly graph was visualized in Bandage 0.8.1 (Wick et al. 2015) to ensure that only fully resolved contigs were used, and read and assembly alignments were also visualized in IGV 2.19.1 (Robinson et al. 2017) to validate the arrangement of contigs, and the resolution of gaps between them. The final assembly was 1,081,996,315 bp long (1,081,979,530 bp of chromosome sequence).

The resolved chromosome-level assembly, which we named Aviagen v1, was further validated by aligning it to the GRCg7b reference genome using nucmer 4.0.0 (Marçais et al. 2018) to generate a whole-genome alignment. This alignment was visualised in a dot plot using the genome alignment viewer 'dot' (https://dot.sandbox.bio/), which showed high collinearity for the genome-genome alignment [Additional file 3, Fig. S1].

Mitochondrion genome

Since the use of CCS mode with PacBio does not allow sequencing circular DNA molecules such as the mitochondrion genome, this genome was obtained from the polished ONT assembly. Briefly, the ONT assembly was mapped against the GRCg7 reference using minimap2 to determine the location of the mitochondrion genome. Since the polished ONT assembly can still contain assembly errors, typically 1-2 bp deletions at homopolymer sequences, variants not supported by the reads (HiFi, ONT and Illumina) were manually corrected with seqkit mutate 2.9.0 (Shen et al. 2016, 2024). A total of eight 1-2 bp indels were corrected this way.

Quality of the assembly

All quality parameters were measured in the primary assembly generated by hifiasm. The contiguity of the assembly was measured via the N50 value using the software quast 5.3.0 (Mikheenko et al. 2018, 2023). This value represents the length of the shortest contig at which 50% of the total assembly length is contained in contigs of this size or longer, and was higher than 28.2 Mb in the raw assembly. In contrast, the ONT assembly was substantially more fragmented (N50 = 2.3 Mb). The percentage of assembly completeness was assessed with the software BUSCO 5.8.2 (Manni et al. 2021) by searching single-copy orthologs against the aves genome database (“-m genome -l aves_odb10”). BUSCO returned 99.3% gene completeness, with only 0.1% fragmented and 0.6% missing orthologs. These quality parameters are higher than the current *Gallus gallus* GRCg7b reference genome (N50 = 18.8 Mb, BUSCO gene completeness = 98.6%). Additionally, we estimated the error rate of the assemblies at the nucleotide level (QV). To do so, we first built a database of k-mers of length 21 bp using the software meryl 1.3 (Rhie et al. 2020) using the Illumina data for this sample. QV was then estimated with merqury 1.3 (Rhie et al. 2020), showing a high genome-wide value of 45 in the Phred scale.

References

Cheng H, Concepcion GT, Feng X, Zhang H, Li H. 2021. Haplotype-resolved de novo assembly using phased assembly graphs with hifiasm. Nat. Methods 18:170-175.

Cheng H, Jarvis ED, Fedrigo O, Koepfli KP, Urban L, et al. 2022. Haplotype-resolved assembly of diploid genomes without parental data. Nat. Biotechnol. 40: 1332–1335.

Cheng H, Asri M, Lucas J, Koren S, Li H. 2024. Scalable telomere-to-telomere assembly for diploid and polyploid genomes with double graph. Nat. Methods 21: 967-970.

Jain C, Koren S, Dilthey A, Phillippy AM, Aluru S. 2018. A fast adaptive algorithm for computing whole-genome homology maps. Bioinformatics 34(17): i748-i756.

Li H. 2018. Minimap2: pairwise alignment for nucleotide sequences. Bioinformatics 34: 3094-3100.

Li H. 2021. New strategies to improve minimap2 alignment accuracy. Bioinformatics 37: 4572-4574.

Marçais G, Delcher AL, Phyllippy AM, Coston R, Salzberg SL et al. 2018. MUMmer 4: a fast and versatile genome alignment system. PLoS Computat. Biol. 14(1): e1005944.

Manni M, Berkeley MR, Seppey M, Simão FA, Zdobnov EM. 2021. BUSCO update: novel and streamlined workflows along with broader and deeper phylogenetic coverage for scoring of eukaryotic, prokaryotic, and viral genomes. Mol. Biol. Evol. 38(10): 4647-4654.

Mikheenko A, Prjibelski A, Saveliev V, Antipov D, Gurevich A. 2018. Versatile genome assembly evaluation with QUAST-LG. Bioinformatics 34(13): i142-150.

Mikheenko A, Saveliev V, Hirsch P, Gurevich A. 2023. WebQUAST: online evaluation of genome assemblies. Nucl. Acids. Res. 51 (W1): W601-W606.

Rhie A, Walenz BP, Koren S, Phyllippy AM. 2020. Merqury: reference-free quality, completeness, and phasing assessment for genome assemblies. Genom. Biol. 21: 245.

Robinson JT, Thorvaldsdóttir H, Wenger AM, Zehir A, Mesirov JP. 2017. Variant review with the Integrative Genomics Viewer (IGV). Cancer Res. 77(21): 31-34.

Shen W, Le S, Hu F. 2016. SeqKit: A cross-platform and ultrafast toolkit for FASTA/Q file manipulation. PLoS ONE 11(10): e0163962.

Shen W, Sipos B, Zhao L. 2024. SeqKit2: A Swiss army knife for sequence and alignment processing. iMeta 3(3): e191.

Walker BJ, Abeel T, Shea T, Priest M, Abouelliel A, et al. 2014. Pilon: an integrated tool for comprehensive microbial variant detection and genome assembly improvement. PLoS ONE: 9(11): e112963.

Wick RR, Schultz MB, Zobel J, Holt KE. 2015. Bandage: interactive visualization of de novo genome assemblies. Bioinformatics 31: 3350-3352.
