# Supplementary material for: The rate and spectrum of germline mutations in chicken from a commercial pedigree line
Source: Genet Sel Evol. 2026 Sep 21;58:70. doi: 10.1186/s12711-026-01085-2 (PMC13591848; doi:10.1186/s12711-026-01085-2)
Supplement: Supplementary file 2 [file 12711_2026_1085_MOESM3_ESM.pdf]

Figure S1. Genome collinearity

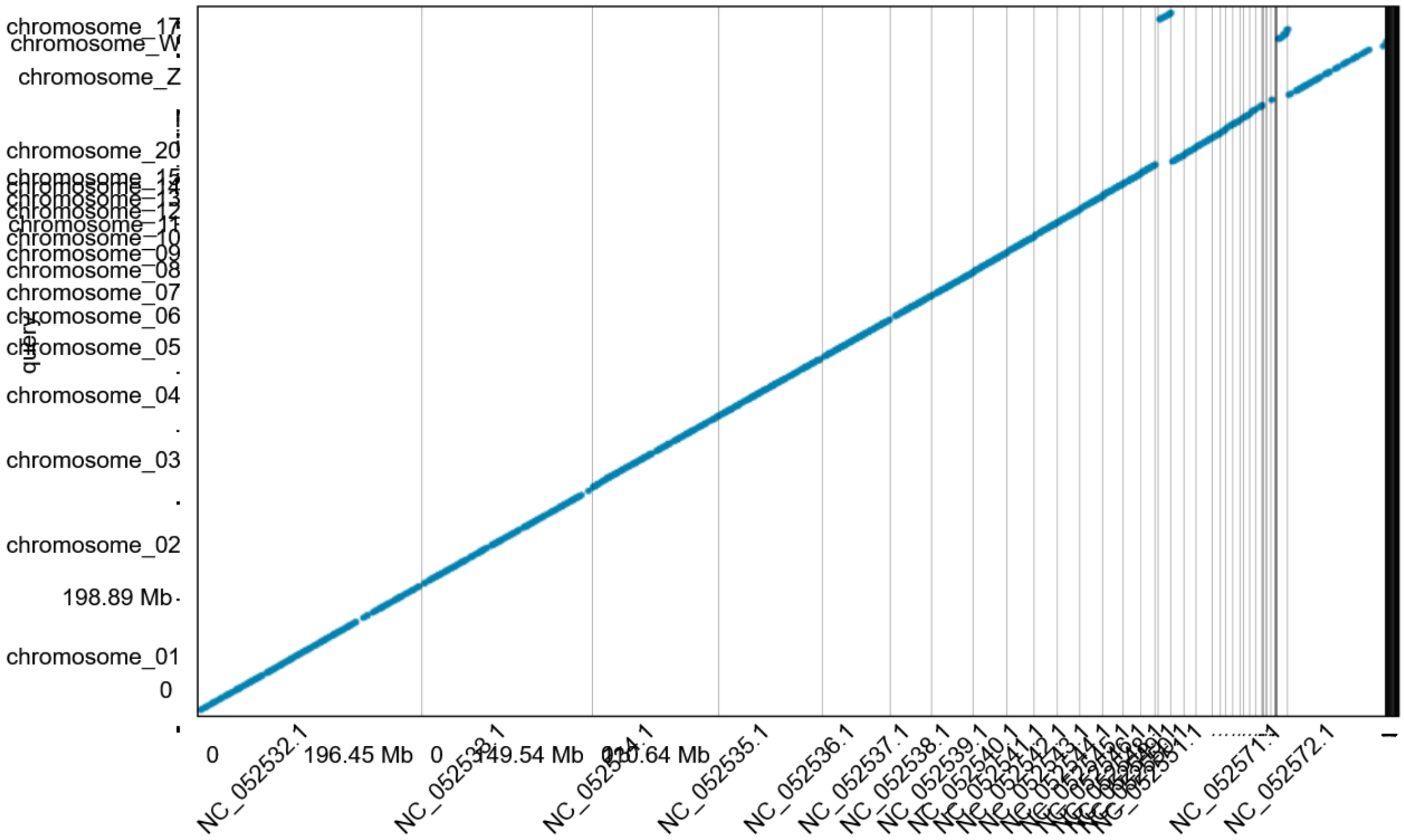

Fig. S1. Dot plot showing the genome-genome alignment between the reference GRCg7b (x-axis) and our de novo Aviagen v1 assembly (y-axis). The alignment includes chromosomes identified by numbers on the label axis, but not all chromosome numbers could be included for visualization, such as most of the microchromosomes and all unmapped contigs. The only two chromosomes that appear out of the central diagonal correspond to Chromosomes 17 and W, which appear with different order in the x and y axes. Unique forward alignments are coloured in blue. Unique reverse alignments (denoting chromosome inversions) are coloured in green, and repetitive alignments are coloured in orange.

Figure S2. Clustered mutations

The large set of SNMs found in our study allows to investigate the extent of mutation clustering from the distribution of inter-mutation distances (IMD), similarly as done before in large mutation accumulation studies (Ness et al. 2015, López-Cortegano et al. 2021). To do so, we generated 1206 synthetic mutations by random sampling coordinates across the callable genome. These simulations were repeated 1000 times. For visualization purposes, Fig. S2 shows the IMD distribution on a decimal logarithmic scale, which highlights the clustering on SNMs within a distance of 100 bp one from each other. This allows to identify eight putative clusters, seven of which occur in the same individual and are therefore considered as genuine SNM clusters. These SNM pairs are in individuals with identities S22 (Chromosome 4: 77,383,994; 77,384,015), S48 (Chromosome 1: 23,218,904; 23,218,947), S124 (Chromosome 4: 28,150,288; 28,150,288), S125 (Chromosome 1: 26,137,436; 26,137,444), S141 (Chromosome 6: 22,135,554; 22,135,559), S148 (Chromosome 2: 79,645,900; 79,645,902), and S182 (Chromosome 9: 20,026,307; 20,026,307).

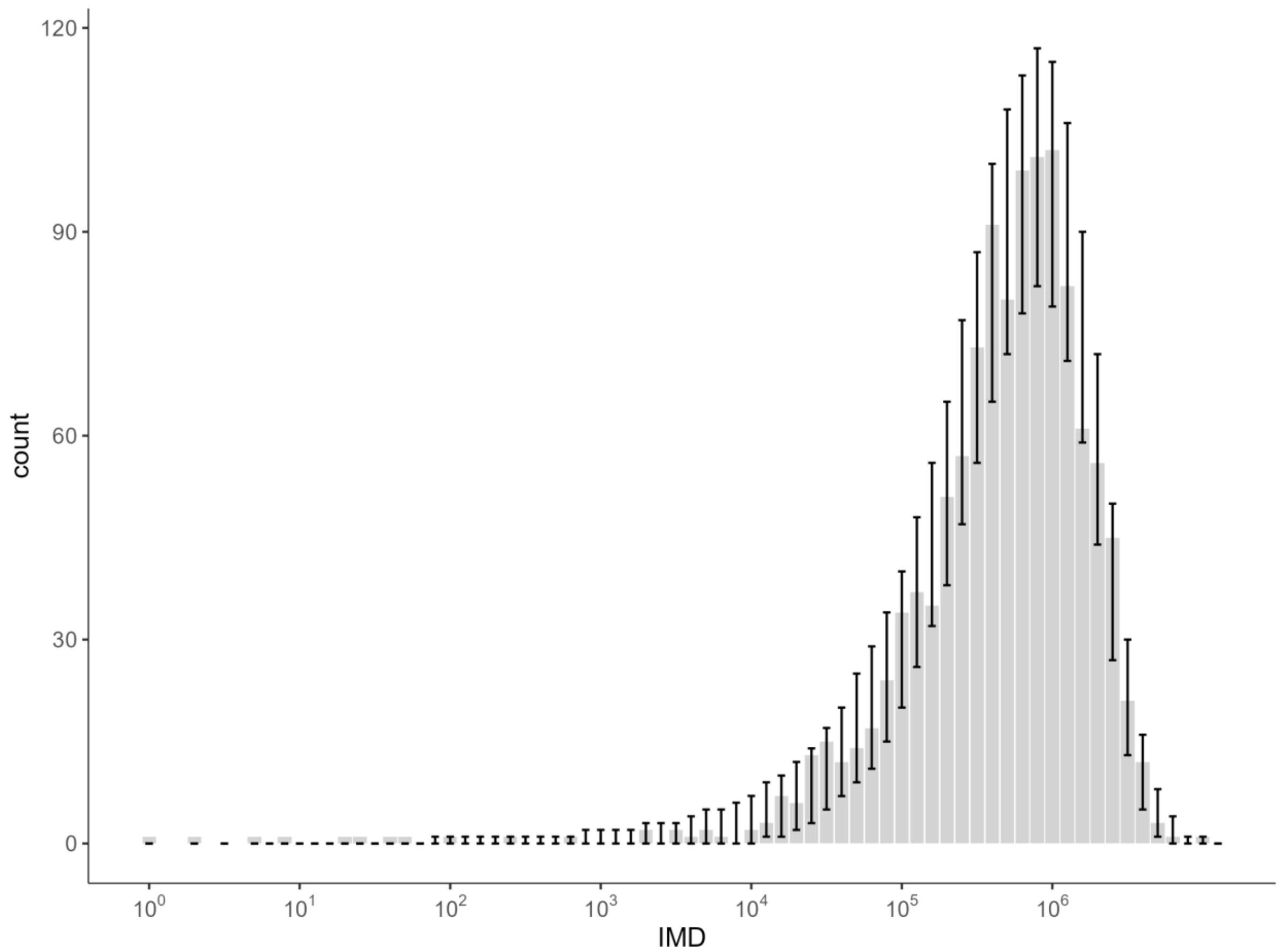

Fig. S2. Distribution of inter-mutation distances (IMD). The observed distribution is shown in grey bars. Black error bars indicate the 95% confidence intervals of the expected distribution obtained from 1000 simulation replicates.

Figure S3. Genome annotation

To provide a functional context for the mutations found in this study, we first generated a genome annotation file by lifting over gene annotation in GFF format from the release 106 of the GRCg7b reference annotation to our chromosome-level assembly. To do so, we used the dedicated tool Liftoff v1.6.3 (Shumate and Salzberg 2021), enabling the dedicated option to polish the annotation ('-polish'). The annotation file did not include explicit genomic coordinates for intros or untranslated regions (UTRs). From the coordinates of genes, exons, and coding sequences (CDSs), introns were inferred as the complementary of genes and exons, and UTRs as the complementary of exons and CDSs.

We then compared the observed distribution of SNMs in the genome to the expected distribution based on random sampling. To do so, we generated synthetic 1206 SNMs using the tools 'random' and 'shuffle' from the BEDTools v2.31.1 (Quinlan and Hall 2010) suite, while ensuring that all SNMs fell within callable sites (see Methods), and then used BEDTools 'intersect' to link each genomic coordinate with its functional annotation. This simulation process was repeated for 1000 replicates. Figure S3 shows a comparison of the observed and expected distribution of SNMs in functional annotations.

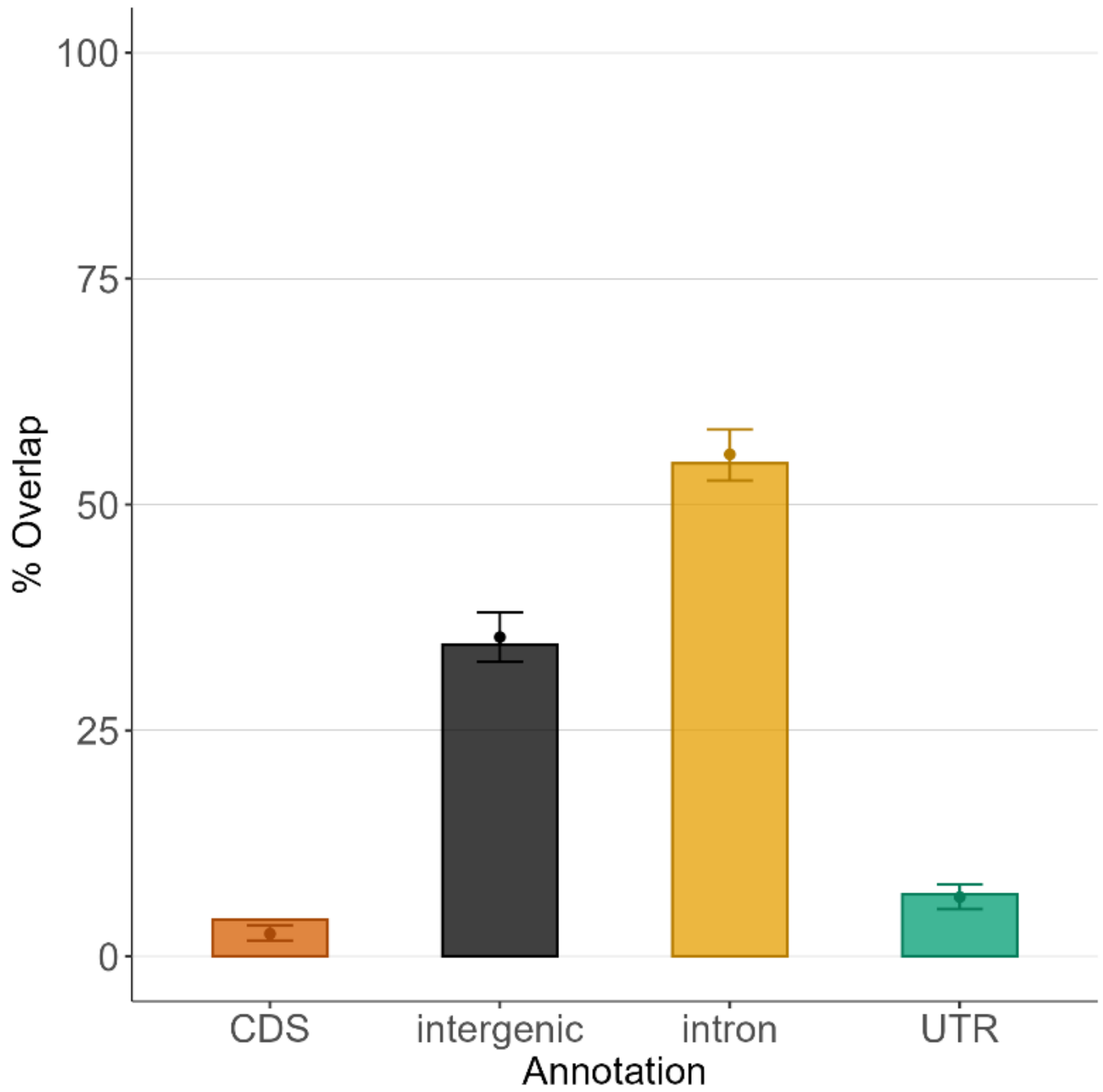

Fig. S3. Distribution of SNMs in functional annotations. In bars, the observed proportion of SNMs overlapping different genomic regions (in colours). In dots with error bars, the median proportions and 95 % confidence intervals for synthetic SNMs, based on 1000 simulation replicates.

Figure S4. SNM spectrum

Figure S4 showed the SNM spectra calculated as in Fig. 2a of the main text, while considering only mutations that could be unambiguously assigned to either male (*N* = 215) or female (*N* = 108) inheritance.

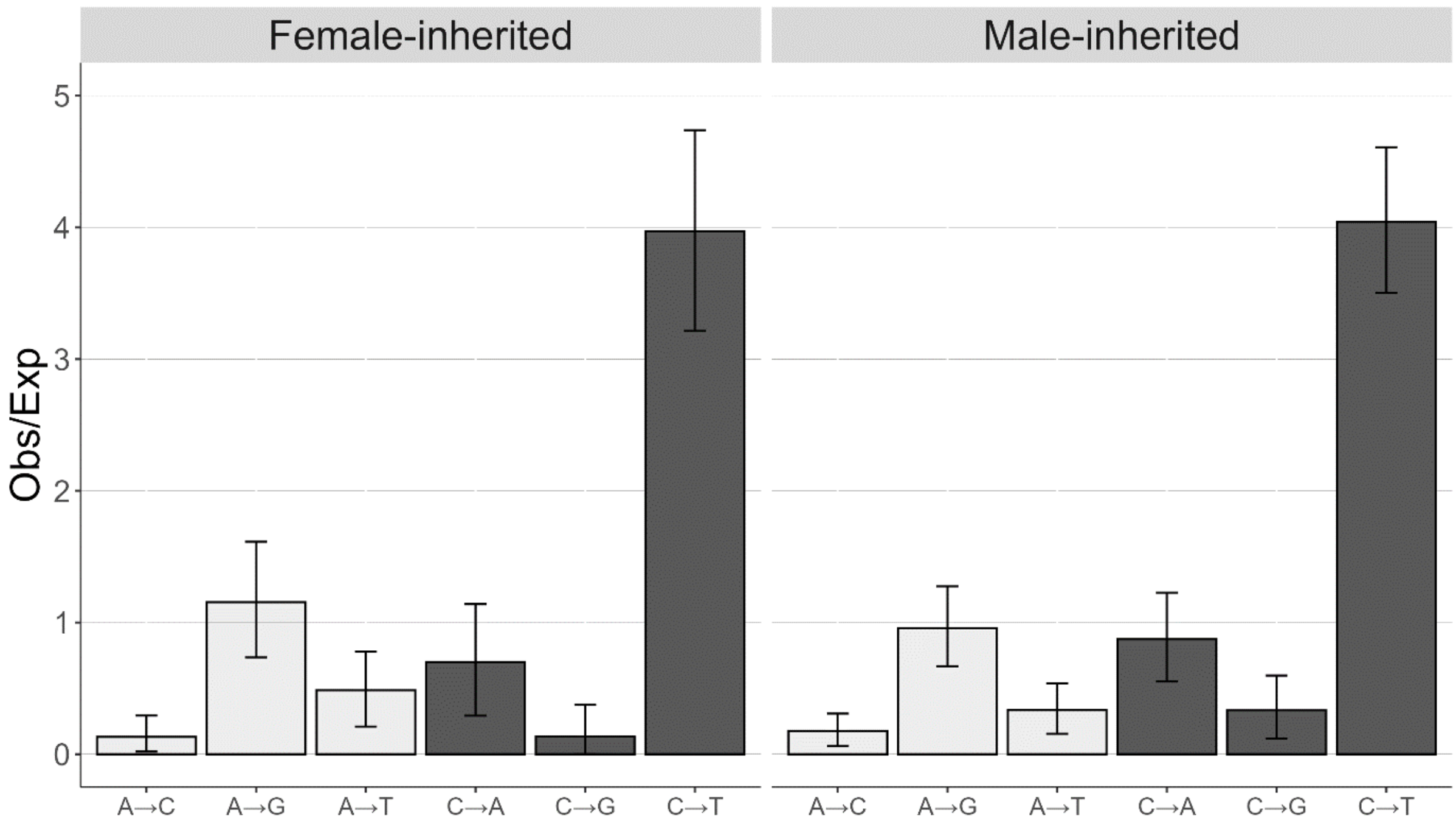

Fig. S4. GC-adjusted ratio of observed vs. expected SNMs. The height of the bars represents the deviation of the observed value from the expectation for all SNMs being equally frequent, after correcting for GC content at callable sites (40.5%). Light bars refer to mutations at A:T sites, whereas dark bars refer to C:G sites. Error bars show 95% confidence intervals based on 1000 bootstrap replicates.

Figures S5-S6. Mutation effects

As noted in the main text, proteins with longer coding sequences (CDS) are more likely to accumulate mutations with codon-altering effects, either missense (moderate effect) or nonsense (high effect) mutations. However, after correcting *PRIME* scores by the CDS length (here, *S* / *L*), longer CDS exhibit intermediate probabilities of such mutations, whereas the shortest peptides show the greatest score variation (Fig. S5). These elevated score variances may partly reflect increased sampling error for amino acids with variable effects in shorter CDS. However, they also suggest that some peptides experience different mutation-driven evolutionary rates and may be under different selection intensities.

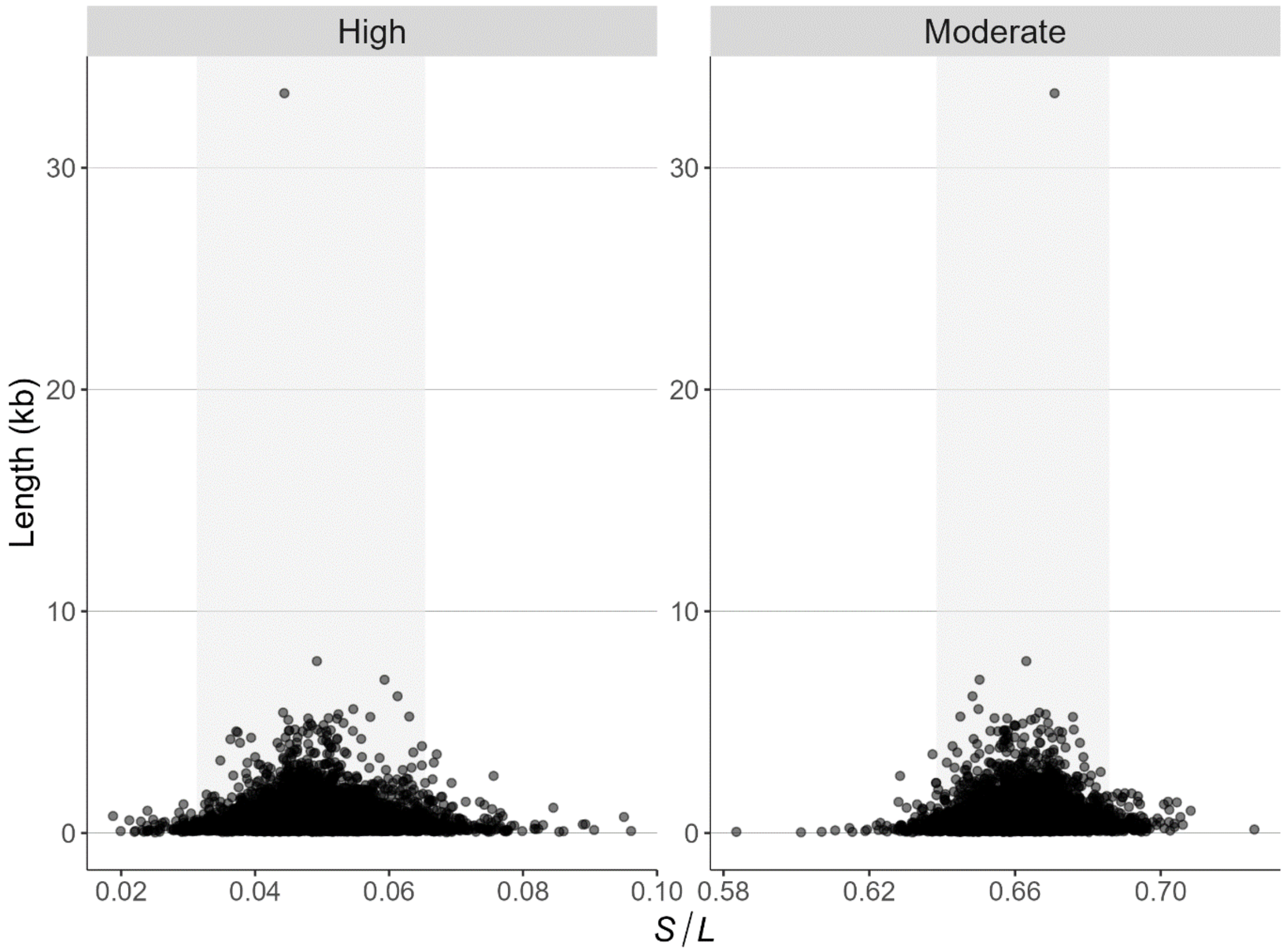

Fig. S5. Association between CDS length (y-axis) and the length-normalized *PRIME* score (*S* / *L*, x-axis) for high (left) and moderate (right) effect mutations. Each dot corresponds to the value *S* / *L* calculated for a single protein sequence. A background grey box delimits 95% CIs for *S* / *L*.

To explore the biological functions most influenced by the influx of new mutations, we arbitrarily selected the genes with upward outlier values for the length-corrected S_H and S_M scores. For these genes, we performed a gene ontology (GO) enrichment analysis with (see Methods). The top 1% GO terms by gene count are shown in Fig. S6. Looking at the results for high-effect mutations, most GO terms were associated with **immunity** (‘regulation of innate immune response’, ‘lymphocyte mediated immunity’, ‘leukocyte mediated immunity’, ‘regulation of immune effector process’, ‘immune response-regulating cell surface receptor signalling pathway’, ‘cell killing’, ‘regulation of lymphocyte mediated immunity’, ‘regulation of leukocyte mediated immunity’, ‘positive regulation of innate immune response’, ‘macroautophagy’ ‘leukocyte mediated cytotoxicity’, ‘innate immune response-activating signalling pathway’, ‘immune response-activating cell surface receptor signalling pathway’, and ‘activation of innage immune response’), and **cellular respiration** (‘mitochondrion organization’, ‘generation of precursor metabolites and energy’, ‘energy derivation by oxidation of organic compounds’, ‘cellular respiration’, and ‘aerobic respiration’). For moderate-effect mutations, the higheststranking GO terms were associated with **organ development and tissue organization** (‘negative regulation of growth’, ‘extracellular structure organization’, ‘extracellular matrix organization’, and ‘external encapsulating structure organization’), followed by abundant GO terms associated with **detoxification** processes (‘response to toxic substance’, ‘detoxification’ and several GO terms associated with cellular response to several metal ions’).

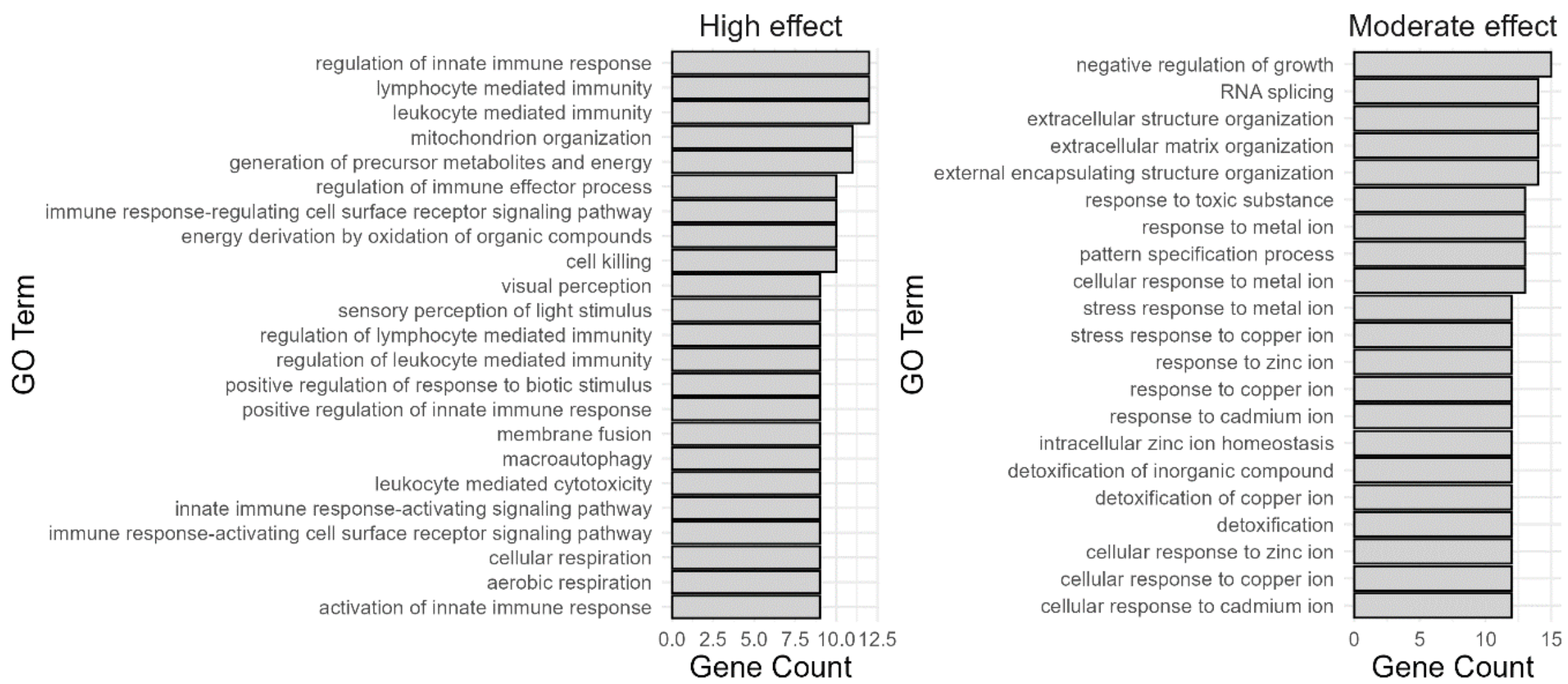

Fig. S6. Top 1% GO terms for CDS with upward outlier length-corrected mutation scores. GO terms are ranked by gene count.

Figure S7. Expected mutation rates at macro- and microchromosomes

Microchromosomes, numbered from 10 to 39, have a substantially higher GC content than macrochromosomes (Fig. 1b). Collectively, we calculated an average GC content of 50.3% for microchromosomes at 144 Mb of callable sites, in contrast to a 40.2% observed in macrochromosomes. Based on the probabilities of mutation at CG and AT sites (Table S2), we can estimate the expected mutation rate for chromosomes based on their GC content (p_{GC}):

$$E(\mu) = p_{GC}\ \mu_{CG} + (1 - p_{GC})\ \mu_{AT}$$

Where $\mu_{CC} = \mu_{C\to A} + \mu_{C\to G} + \mu_{C\to T}$ and $\mu_{AT} = \mu_{A\to C} + \mu_{A\to G} + \mu_{A\to T}$ using values from Table S2. Using data for the GC content at callable sites, we estimate expected mutation rates of μ = 3.56×10^{-9} for macrochromosomes, and μ = 3.97×10^{-9} for microchromosomes. Figure S7a shows the per-chromosome GC content at callable sites and their corresponding values of expected mutation rates.

To explore the effect of mutations on microchromosomes, we repeated simulations introducing synthetic mutations as described in the main text. This resulted in a spectrum of SNMs with different effect types as in Fig. S7b, a figure analogous to Fig. 2b in the main text for synthetic mutations genome-wide. As result, we observed an overall increase in the proportion of low effect mutations in microchromosomes compared to what observed genome-wide (31.1% vs. 28.8%), together with a decrease in moderate (64.3% vs. 65.7%) and high (4.6% vs. 5.4%) effect mutations.

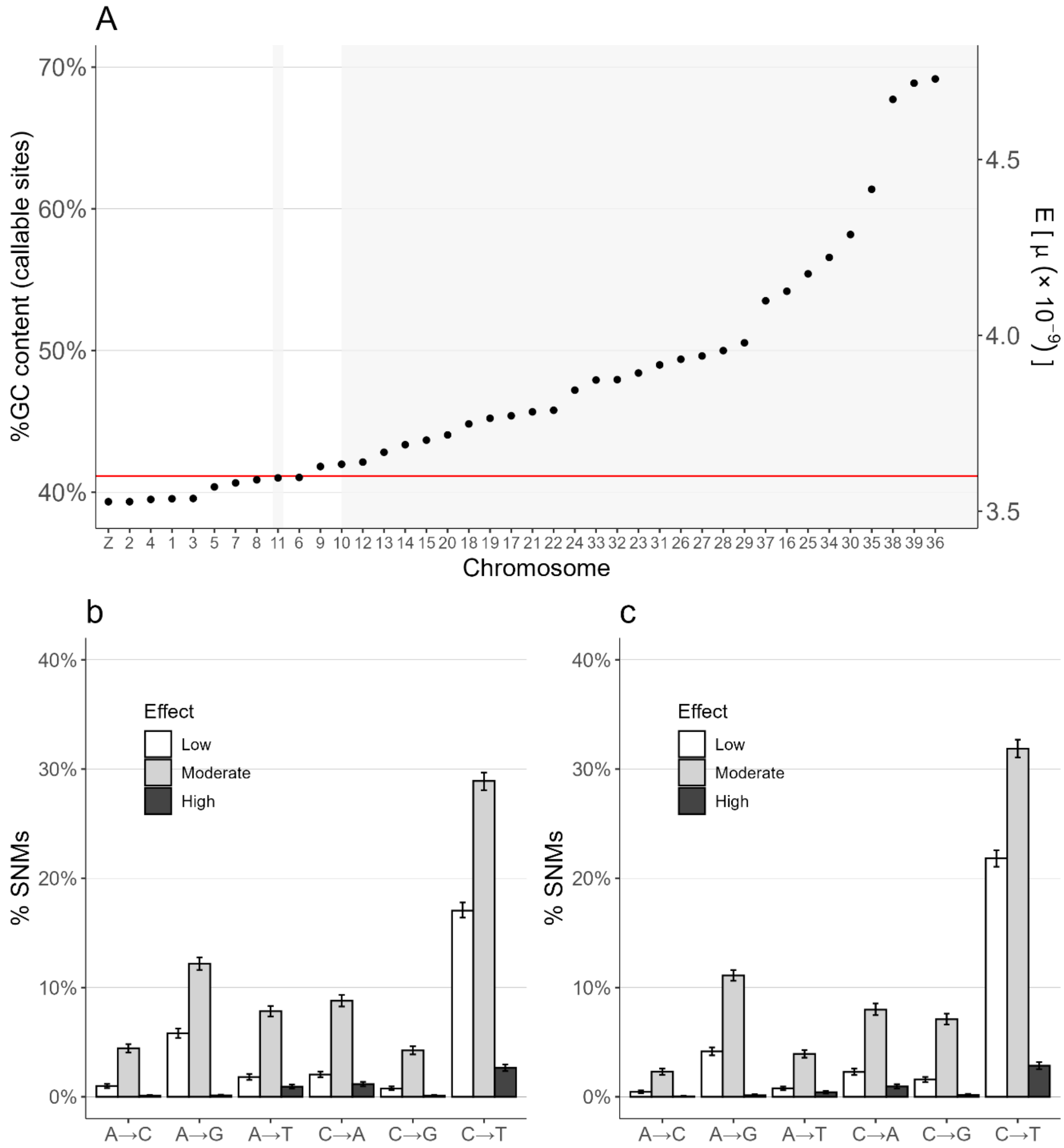

Fig. S7. Mutations per chromosome type. a) % GC content (left) and expected mutation rates (right) per chromosome. Chromosomes are sorted by GC content. A light grey background highlights microchromosomes. A horizontal red line highlights the average genomic value 3.6×10^{-9}. b) Proportion of SNMs predicted to have low, moderate or high effect sizes in macrochromosomes, based on computer simulations. Error bars show 95 % CIs based on 1000 simulation replicates. c) Similar analysis as in b) using microchromosome data.

Figure S8. Sequence context effect on SNM rates

Sequence context is known to influence SNM rates. Using methods similar to López-Cortegano et al. (2024), we examined the effect of trinucleotide context on SNM rates in chicken. In short, each SNM was assigned to its possible trinucleotide contexts, and mutation rates per context were subsequently calculated from the count of mutations divided by two times the count of that context at callable sites. Figure S8 shows that SNM rates vary substantially across trinucleotide contexts, and highlights a pronounced effect for (methylated) CpG sites, particularly for C→T transitions.

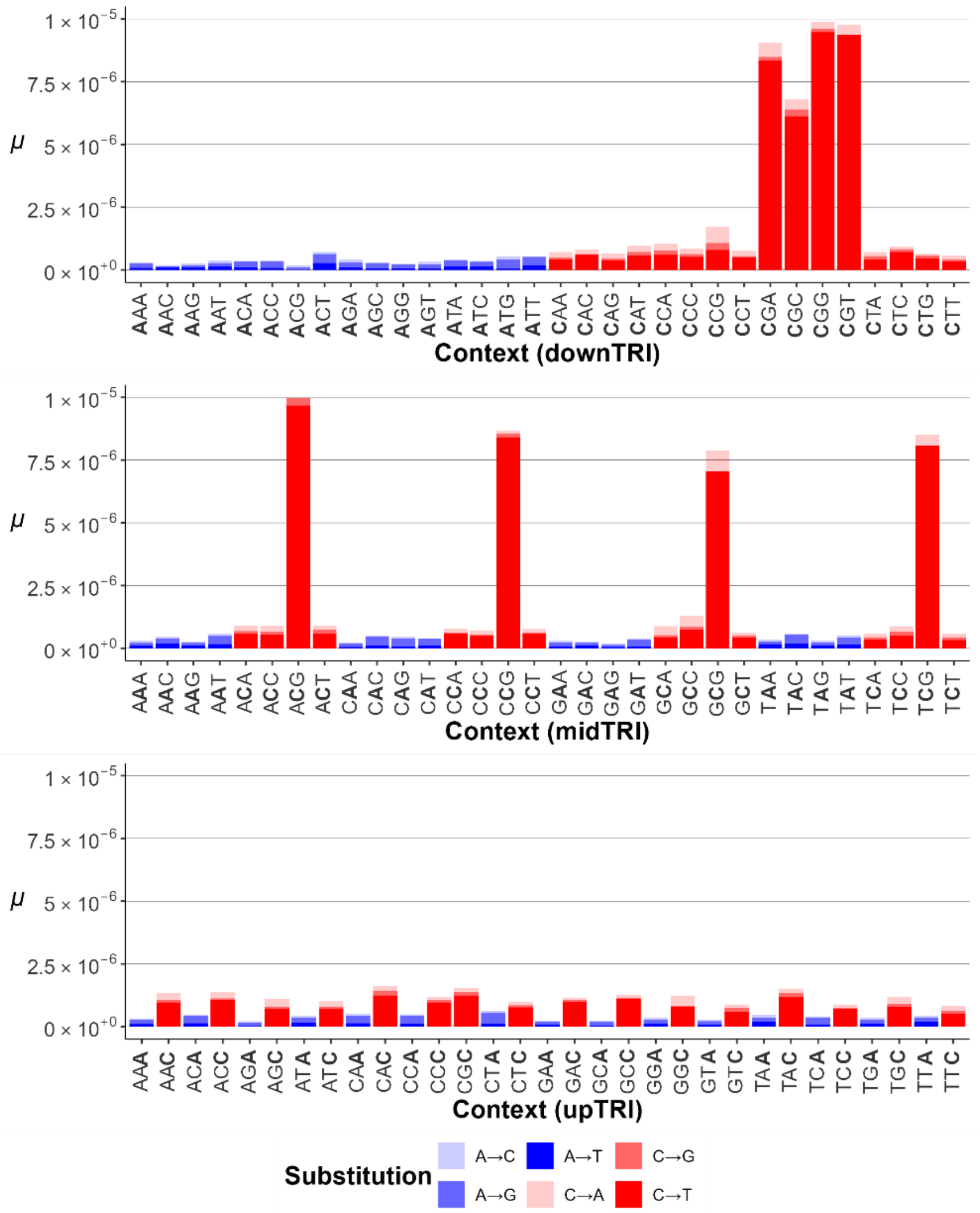

Fig. S8. Spectrum of SNMs across different trinucleotide sequence contexts, with the mutation rate on the y-axis and the trinucleotide context on the x-axis. Trinucleotide contexts are 5→'3' oriented, and the nucleotide marked with bold text in the legend identifies the mutated site. The figure comprises three panels: the top panel (downTRI) shows the downstream context where the mutated site is followed by two nucleotides, the middle panel (midTRI) shows the full surrounding trinucleotide sequence with one nucleotide on each side of the mutated site, and the bottom panel (upTRI) shows the upstream context where the mutated site is preceded by two nucleotides. G/C mutated sites are marked in red, and A/T mutated sites in blue, with different SNM types represented by varying shades of the same colour within each site type.

References

López-Cortegano E, Craig RJ, Chebib J, Samuels T, Morgan AD, et al. 2021. De novo mutation rate variation and its determinants in Chlamydomonas. Mol. Biol. Evol. 38(9): 3709-3723.

López-Cortegano E, Chebib J, Jonas A, Vock A, Künzel S, et al. 2024. Variation in the spectrum of new mutations among inbred strains of mice. Mol. Biol. Evol. 41(8): msae163.

Ness RW, Morgan AD, Vasanthakrishnan RB, Colegrave N, Keightley PD. 2015. Extensive de novo mutation rate variation between individuals across the genome of Chlamydomonas reinhardtii. Genome Res. 25(11): 1739-1749.

Quinlan AR, Hall IM. 2010. BEDTools: a flexible suite of utilities for comparing genomic features. Bioinformatics 26(6): 841-842.

Shumate A, Salzberg SL. 2021. Liftoff: accurate mapping of gene annotations. Bioinformatics. 37(12): 1639-1643.
